# Laying the Foundation-Stone of the Children's Convalescent Home, Bognor

**Published:** 1897-11-06

**Authors:** 


### LAYING THE FOUNDATION-STONE OF THE CHILDREN'S CONVALESCENT HOME, BOGNOR.

Barring a somewhat thick fog in London on the morning of Thursday, October 28th, nothing marred the pleasures of the company assembled to witness the laying of the foundation-stone of the convalescent home to be built in connection with the East London Hospital for Children, Shadwell, E. The London guests left Victoria in special carriages attached to the 10.30 train, and were soon whirled into a country beautiful with sunshine and autumn colours. "Dear little Bognor," as her Majesty calls it in memory of a happy visit in her youth, appeared to its greatest advantage, the clean, clear air contrasting most agreeably with the fog left behind. The visitors joined the local guests at the site in Aldwick Road. A few flags and a scrap of red bunting, bearing the word "Welcome," nailed across the entrance, were the only attempts at decorations, though the skeleton framework had been put up for an awning in case of rain. When preparations for the ceremony were complete, a photograph was taken. The Rev. W. B. Bladon, M.A., of St. John the Baptist, Bognor, conducted a short service, concluding with a benediction. In consequence of Mr. T. D. Galpin's indisposition, the stone was laid by Mr. H.W. Trinder, who, after expressing his own and colleague's regret at the absence of Mr. Galpin, gave a short description of the work of the parent hospital for the benefit of the local visitors. The Right Hon. Viscount Falkland (vice-chairman of the Board of Management) proposed the vote of thanks to Mr. Trinder. An excellent lunch was served at Victoria Mansions, West, to which about fifty persons sat down. The toasts given were "The Queen," "The Prosperity of the Convalescent Home," by Mr. H. W. Trinder, and "The Visitors," by Mr. George Russell, to which Mr. Herbert N. Travers (chairman of the Bognor Urban District Council) replied. After an hour by the sea, which was as still as on a summer's day, the return party left for town at 5.20 p.m.

The estimated cost of the new home is £4,500, of which sum £3,000 is already in hand, Mr. T. D. Galpin having been a liberal donor. It will accommodate twenty-two children, a nursing staff, and servants. On the entrance floor will be the dining hall, play-room, kitchen, lift, &c., and two wards of four beds each for bad surgical cases. On the first floor the matron's bed and sitting rooms, the nurses' sitting-room, two wards of ten beds each, one for boys and one for girls ; and on the top floor the nurses and servants' bedrooms. The foundations are well laid, the outer walls double, and all have slabs of slate inserted about two feet from the ground. A wide verandah will stretch across the entrance from wing to wing, whilst a spacious playground will be laid out on the ground now a wilderness of the bricklayer.

Many reasons combine to make a convalescent home desirable, and to point out Bognor as the most desirable locality for the new home. In the first place, the hospital authorities found it expensive, and not altogether satisfactory, to board out their convalescents, as their own medical men could not watch the progress of the little patients. The sites taken into consideration on the East Coast were so exposed as to necessitate closing the home during three months of the year, whilst in Bognor the home can be kept open all the year round. In addition, the safety of the shore was a great attraction. The new home faces the sea, but stands some distance back from it, a stretch of green sward lying between. Additional ground has been acquired in the rear, which will permit of the home being enlarged when the need arises.

A committee of ladies of the neighbourhood will be invited to supervise the institution which will be in charge of the matron and a staff of probably five nurses.

